# Tumor hepatitis B virus RNA identifies a clinically and molecularly distinct subset of hepatocellular carcinoma

**DOI:** 10.1371/journal.pcbi.1008699

**Published:** 2021-02-09

**Authors:** Huat Chye Lim, John D. Gordan

**Affiliations:** Division of Hematology and Oncology, University of California, San Francisco, San Francisco, California, United States of America; University of Cambridge, UNITED KINGDOM

## Abstract

Hepatitis B virus (HBV) infection contributes to hepatocellular carcinoma (HCC) initiation and is associated with worse outcomes. Many prior studies of HBV-related HCC have not accounted for potential heterogeneity among HBV-related tumors by assessing whether HBV activity is present in tumor tissue. Here, we measured tumor HBV RNA, a proxy for viral activity, and investigated the association between HBV RNA status and several clinicogenomic characteristics. We obtained clinical, mutation, RNA-Seq and survival data for 439 HCC tumors from The Cancer Genome Atlas and International Cancer Genome Consortium. Tumors were classified as HBV RNA positive if they harbored >1 HBV RNA read per million human reads. We investigated the association between HBV RNA status and nonsynonymous somatic mutations, gene set expression, homologous recombination deficiency (HRD) score and mutation-specific survival. HBV RNA positive status was associated with higher nonsynonymous mutation rates of multiple genes, including *TP53* and *CDKN2A*, while HBV RNA negative status was associated with higher nonsynonymous *BAP1* mutation rate. HBV RNA positive status was also associated with increased transcription of genes involved in multiple DNA damage repair pathways, genes upregulated by *MYC* and mTORC1, and genes overexpressed in several HCC subclasses associated with a proliferative phenotype. Further, HBV RNA positive status was associated with increased three-biomarker HRD score (22.2 for HBV RNA+ vs. 16.0 for HBV RNA-). Finally, HBV RNA status was associated with multiple mutation-specific survival differences, including decreased survival for HBV RNA positive patients with nonsynonymous *KEAP1* mutations compared to those without (hazard ratio 4.26). HCC tumors harboring genomic evidence of HBV activity therefore constitute a distinct HCC subset characterized by specific differences in nonsynonymous mutations, gene set expression, three-biomarker HRD score and mutation-specific survival.

## Author summary

Hepatocellular carcinoma, the most common type of liver cancer, is the second leading cause of cancer death worldwide and is most commonly caused by hepatitis B virus infection. Currently, scientists have an incomplete understanding of the genomic basis of hepatocellular carcinoma associated with hepatitis B virus infection, because prior studies have been limited by imprecision in assessing hepatitis B virus infection status and heterogeneity in hepatitis B virus activity levels in liver tumors. This has limited scientists’ ability to devise new diagnostic and therapeutic options for hepatocellular carcinoma. In this study, we used computational genomics to directly measure hepatitis B virus RNA levels in a large dataset of hepatocellular carcinoma tumors, and found that tumors with measurable hepatitis B virus RNA levels are associated with a specific set of clinical and genomic characteristics. These characteristics have not previously been reported and harbor implications for future clinical and genomics research in hepatocellular carcinoma, as well as computational genomics efforts in other cancer types.

## Introduction

Hepatocellular carcinoma (HCC) is the sixth most incident cancer and second most common cause of cancer death worldwide [1]. Most cases occur in the setting of preexisting liver disease, most commonly hepatitis B virus (HBV) infection, which is thought to be responsible for over half of HCC cases worldwide [2]. In contrast to many other cancers, liver cancer mortality has risen in recent years, with a 43% increase in age-adjusted death rate in the United States between 2000 and 2016 [3]. Although our genomic understanding and the available systemic therapy options for HCC have grown in recent years, both remain relatively limited and somewhat disconnected. Thus, an unmet need exists to better understand HCC genomics, particularly with respect to risk factors such as HBV infection, in order to better prevent and treat this disease.

HBV is a small enveloped DNA virus with a partially double-stranded genome that persists in and can transform infected hepatocytes via the formation and integration of episomal covalently closed circular DNA [4]. It affects approximately 5% of the global population and is thought to promote tumorigenesis by increasing chromosomal instability and cell proliferation [5]. HBV-related HCC has been associated with several clinical differences relative to other HCC, including associations with male gender, Asian ethnicity, younger age at initial diagnosis and less preexisting cirrhosis [6,7].

Several recent studies have advanced our understanding of how HBV contributes to HCC tumorigenesis at the genomic level. HBV-related HCC has been associated with specific oncogenic genomic events, including *TERT* alterations (e.g., HBV DNA integration into the *TERT* promoter appears to promote telomerase overexpression and cell immortality) [8] and *TP53* promoter repression (leading to reduced TP53 expression) [9]. Sites of recurrent HBV integration into the genome have been characterized, including areas near the cell cycle genes *CCNA2* and *CCNE1* and the tumor suppressor gene *KMT2D* [6,10]. Furthermore, HBV infection is known to induce epigenetic changes that promote tumorigenesis, including changes in DNA methylation, histone modification and microRNA expression, some of which are thought to be mediated by the HBx protein [11–14]. Finally, HBV infection can synergistically increase the risk of tumorigenesis when other HCC risk factors are present, such as exposure to aflatoxin B1 (AFB1), a mycotoxin present in subtropical Asia and Africa. AFB1 is metabolized in the liver to a mutagen that promotes the *TP53 R249S* mutation, and exposure to both it and HBV increases the risk of HCC incidence by severalfold compared to either risk factor alone [15].

Despite these recent advances, many prior studies of HBV-related HCC have not accounted for potential genomic and molecular heterogeneity among HBV-related HCC tumors. A prime example of this is the fact that HBV-related HCC tumors may or may not harbor HBV activity in tumor tissue (e.g., viral activity may be absent in a tumor from a patient whose HBV infection cleared spontaneously or in response to antiviral therapy prior to tissue sampling). We hypothesized that direct HBV RNA measurement in tumor tissue, as a proxy for viral activity, could lead to more nuanced evaluation of tumor HBV status and thus to expanded analysis of HCC genomic datasets with respect to HBV infection. Here, we report that tumor HBV RNA measurement is associated with a distinct pattern of differentially mutated genes, differentially expressed gene sets and differences in homologous recombination deficiency and mutation-specific survival.

## Results

We examined 439 HCC tumors, of which 124 were HBV RNA positive and 315 were HBV RNA negative. Consistent with prior studies of HBV-related HCC, we found that HBV RNA positive status was associated with male gender, younger age and higher tumor Edmondson grade at diagnosis (*P* = 0.0006 or less for all; Table 1) [7]. As expected, it was also associated with a clinical history of hepatitis B and no clinical history of hepatitis C. We found no association with tumor stage at diagnosis, tumor vascular invasion or clinical history of alcohol consumption. Of note, in The Cancer Genome Atlas (TCGA) dataset, there was excellent concordance between HBV RNA status and genomic evidence of HBV integration in tumor tissue (*P* < 0.0001; S1 Table), and between HBV RNA status in tumor and paired normal liver tissue for the 50 TCGA tumors for which genomic data from paired normal tissue were available (*P* < 0.0001; S2 Table).

**Table 1:** Clinical, histologic and pathologic characteristics for the 439 HCC cases in the combined TCGA and ICGC (LIRI-JP) cohort. Pathologic stage was determined using either AJCC (for TCGA) or LCSGJ (for ICGC) criteria. Vascular invasion includes both macrovascular and microvascular invasion, where known (whether invasion was macrovascular or microvascular was available for TCGA cases, but not for ICGC cases). For Edmondson grade, ICGC cases annotated as “grade I-II” were counted as grade II and cases annotated as “grade II-III” were counted as grade III. *p* values obtained from χ^2^ (*) and Mann-Whitney (**) tests are shown. TCGA, The Cancer Genome Atlas; ICGC, International Cancer Genome Consortium; AJCC, American Joint Committee on Cancer; LCSGJ, Liver Cancer Study Group of Japan; NS, not significant.

			HBV RNA+	HBV RNA-	*p*
**Total patients**			124	315	N/A
**Cohort**	TCGA		100 (81%)	271 (86%)	N/A
	ICGC (LIRI-JP)		24 (19%)	44 (14%)	
**Gender**	Male		100 (81%)	201 (64%)	0.0006 (*)
	Female		24 (19%)	114 (36%)	
**Age at diagnosis**	Mean ± SD		54.2 ± 12.0	62.9 ± 13.1	< 0.0001 (**)
**Risk factors**	HBV clinical history		87 (70%)	44 (14%)	< 0.0001 (*)
	HCV clinical history		2 (2%)	79 (25%)	< 0.0001 (*)
	Alcohol consumption		45 (36%)	106 (34%)	NS
**Edmondson grade at diagnosis**	Grade I		12 (10%)	47 (15%)	0.0003 (*)
	Grade II		50 (40%)	161 (51%)	
	Grade III		51 (41%)	83 (26%)	
	Grade IV		8 (6%)	4 (1%)	
	Unknown		3 (2%)	20 (6%)	
**Pathologic stage at diagnosis**	Stage I		54 (44%)	129 (41%)	NS
	Stage II		28 (23%)	84 (27%)	
	Stage III		38 (31%)	73 (23%)	
	Stage IV		3 (2%)	10 (3%)	
	Unknown		1 (1%)	19 (6%)	
**Vascular invasion**	Present		37 (30%)	105 (33%)	NS
	Absent		65 (52%)	176 (56%)	
	Unknown		22 (18%)	34 (11%)	

**Fig 1:**
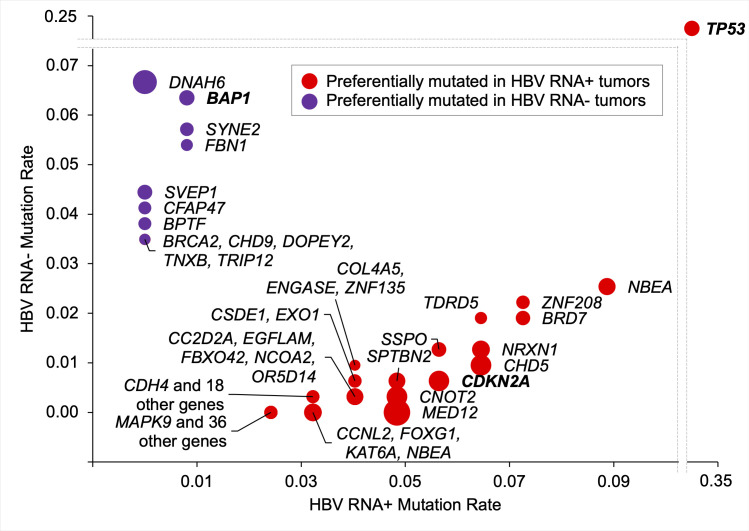
Genes where nonsynonymous somatic mutation rate depended significantly on HBV RNA status in the combined TCGA and ICGC cohort. Circle size is inversely proportional to log(*p*) value. Genes shown in bold are among the 26 significantly-mutated genes identified in the TCGA hepatocellular carcinoma study. HBV, hepatitis B virus; TCGA, The Cancer Genome Atlas; ICGC, International Cancer Genome Consortium.

**Fig 2:**
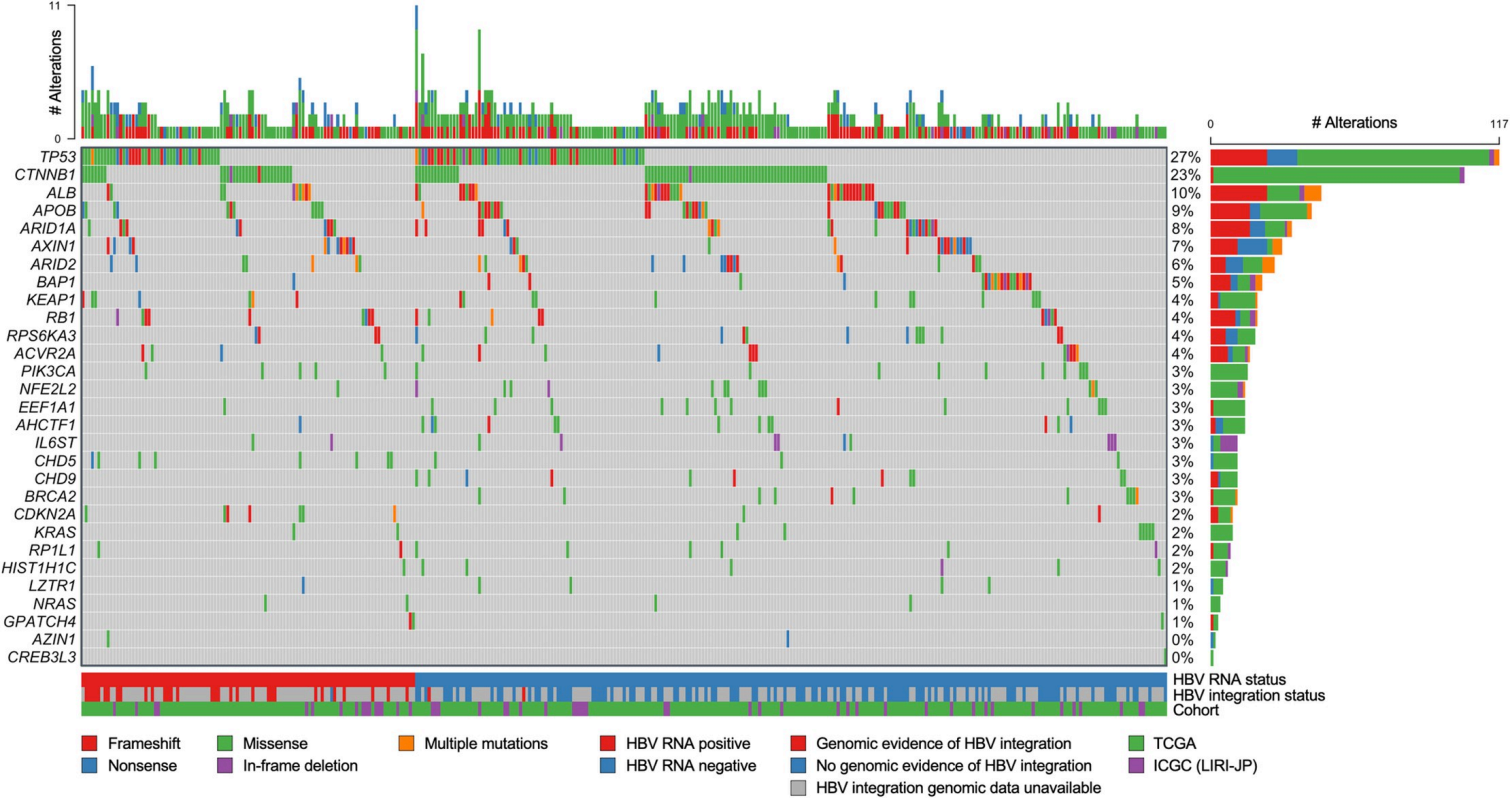
Nonsynonymous somatic mutational landscape of 29 selected genes in the combined TCGA and ICGC cohort, including the 26 significantly-mutated genes identified in the TCGA hepatocellular carcinoma study. Top panel shows number of nonsynonymous somatic mutations per subject. Right panel shows number of nonsynonymous somatic mutations per gene. Cohort, mutation type, HBV RNA status and status of genomic evidence of HBV integration are indicated in legend at bottom. HBV, hepatitis B virus; TCGA, The Cancer Genome Atlas; ICGC, International Cancer Genome Consortium.

We then examined the effect of HBV RNA status on the tumor somatic mutation spectrum and found that the nonsynonymous somatic mutation rates of 93 genes depended significantly on HBV RNA status (Figs 1 and 2 and Table 2). These included the tumor suppressor genes *TP53*, *CDKN2A* and *CHD5*, which were more frequently mutated in HBV RNA positive tumors (*P* < 0.02 for all), as well as the tumor suppressor gene *BAP1*, the chromatin remodeling gene *CHD9* and the DNA damage repair gene *BRCA2*, which were more frequently mutated in HBV RNA negative tumors (*P* < 0.04 for all). *TP53*, *CDKN2A* and *BAP1* are noteworthy for being among the 26 significantly mutated genes identified in the TCGA HCC genomics study [6]. We also found, consistent with prior studies, that *TP53* had a substantially higher mutation rate compared to all other differentially mutated genes, and a significantly higher mutation rate in HBV RNA positive tumors [6,7]. Strikingly, in our cohort, all 11 *BRCA2* mutations, all 11 *CHD9* mutations and 20 of the 21 *BAP1* mutations identified were present in HBV RNA negative tumors; HBV RNA positive tumors harbored no *BRCA2* or *CHD9* mutations and only one *BAP1* mutation. Finally, we found that most differentially mutated genes (82 of 93) were preferentially mutated in HBV RNA positive tumors.

**Table 2:** Nonsynonymous somatic mutation rates by HBV RNA status for 29 selected genes in the combined TCGA and ICGC cohort, including the 26 significantly-mutated genes identified in the TCGA hepatocellular carcinoma study. Bolded genes are those where nonsynonymous somatic mutation rate depended significantly on HBV RNA status. *p* values obtained from Fisher’s exact test are shown. HBV, hepatitis B virus; TCGA, The Cancer Genome Atlas; ICGC, International Cancer Genome Consortium; NS, not significant.

Gene	Total Mutations	HBV RNA+ Mutation Rate	HBV RNA- Mutation Rate	*p*
***TP53***	**117**	**35% (44/124)**	**23% (73/315)**	**0.012**
*CTNNB1*	103	25% (31/124)	23% (72/315)	NS
*ALB*	45	8% (10/124)	11% (35/315)	NS
*APOB*	41	9% (11/124)	10% (30/315)	NS
*ARID1A*	33	8% (10/124)	7% (23/315)	NS
*AXIN1*	29	10% (12/124)	5% (17/315)	NS
*ARID2*	26	6% (7/124)	6% (19/315)	NS
***BAP1***	**21**	**1% (1/124)**	**6% (20/315)**	**0.012**
*KEAP1*	19	6% (7/124)	4% (12/315)	NS
*RB1*	19	7% (9/124)	3% (10/315)	NS
*RPS6KA3*	18	3% (4/124)	4% (14/315)	NS
*ACVR2A*	16	3% (4/124)	4% (12/315)	NS
*PIK3CA*	15	4% (5/124)	3% (10/315)	NS
*AHCTF1*	14	1% (1/124)	4% (13/315)	NS
*EEF1A1*	14	1% (1/124)	4% (13/315)	NS
*NFE2L2*	14	1% (1/124)	4% (13/315)	NS
***BRCA2***	**11**	**0% (0/124)**	**3% (11/315)**	**0.039**
***CHD5***	**11**	**6% (8/124)**	**1% (3/315)**	**0.002**
***CHD9***	**11**	**0% (0/124)**	**3% (11/315)**	**0.039**
*IL6ST*	11	2% (2/124)	3% (9/315)	NS
***CDKN2A***	**9**	**6% (7/124)**	**1% (2/315)**	**0.003**
*KRAS*	9	2% (2/124)	2% (7/315)	NS
*RP1L1*	8	2% (2/124)	2% (6/315)	NS
*HIST1H1C*	7	1% (1/124)	2% (6/315)	NS
*LZTR1*	5	1% (1/124)	1% (4/315)	NS
*NRAS*	4	2% (2/124)	1% (2/315)	NS
*GPATCH4*	3	2% (2/124)	0% (1/315)	NS
*AZIN1*	2	1% (1/124)	0% (1/315)	NS
*CREB3L3*	1	0% (0/124)	0% (1/315)	NS

Several genes where we found no significant difference in nonsynonymous somatic mutation rate based on HBV RNA status are nonetheless worth noting. These include *NFE2L2*, which encodes NRF2, a transcription factor involved in the oxidative stress response pathway, and in which mutations have previously been reported as present only in non-HBV HCC [7]. Although we also found a trend towards *NFE2L2* being more frequently mutated in HBV RNA negative tumors, which harbored all except one of the 14 *NFE2L2* mutations present in our cohort, this finding did not reach the threshold for significance (*P* = 0.13; Table 2). Additionally, consistent with prior findings, in our cohort, *ARID1A*, *ARID2* and *PIK3CA* mutation rates did not vary significantly by HBV RNA status [7]. Finally, despite prior findings that mutations in the Wnt/β-catenin pathway genes *AXIN1* and *CTNNB1* are more frequently present in HBV-related HCC [7,16], in our cohort, *AXIN1* and *CTNNB1* mutation rates did not vary significantly by HBV RNA status.

To evaluate the genomic overlap between HBV RNA positive tumors and tumors that were clinically annotated as HBV-related, we then examined the relationship between HBV clinical annotation and nonsynonymous somatic mutations for tumors in the TCGA dataset (for which this annotation data were available). We categorized patients as HBV annotation positive if they were annotated as having a clinical history of either hepatitis B or hepatitis B surface antigen positivity, and as HBV annotation negative if they were annotated as having a clinical history of neither. There were 225 HBV annotation positive tumors and 88 HBV annotation negative tumors; the remaining tumors had insufficient annotation data for categorization. We found that the nonsynonymous somatic mutation rates of 37 genes depended significantly on HBV annotation status, but strikingly, of these 37 genes, only one, *CFAP47*, was also among the 93 genes whose mutation rate depended significantly on HBV RNA status. (In contrast, in the core TCGA dataset, where complete integration analysis was available, the nonsynonymous somatic mutation rates of 109 genes depended significantly on whether genomic evidence of HBV integration was present, of which 90 overlapped with those genes whose mutation rate depended significantly on HBV RNA status.) Additionally, while the vast majority of HBV RNA positive tumors (93 of 100) were also HBV annotation positive, the distribution of HBV annotation status among HBV RNA negative tumors was more varied: 132 (48.8%) were HBV annotation positive and 86 (31.7%) were HBV annotation negative (S1 Table).

**Fig 3:**
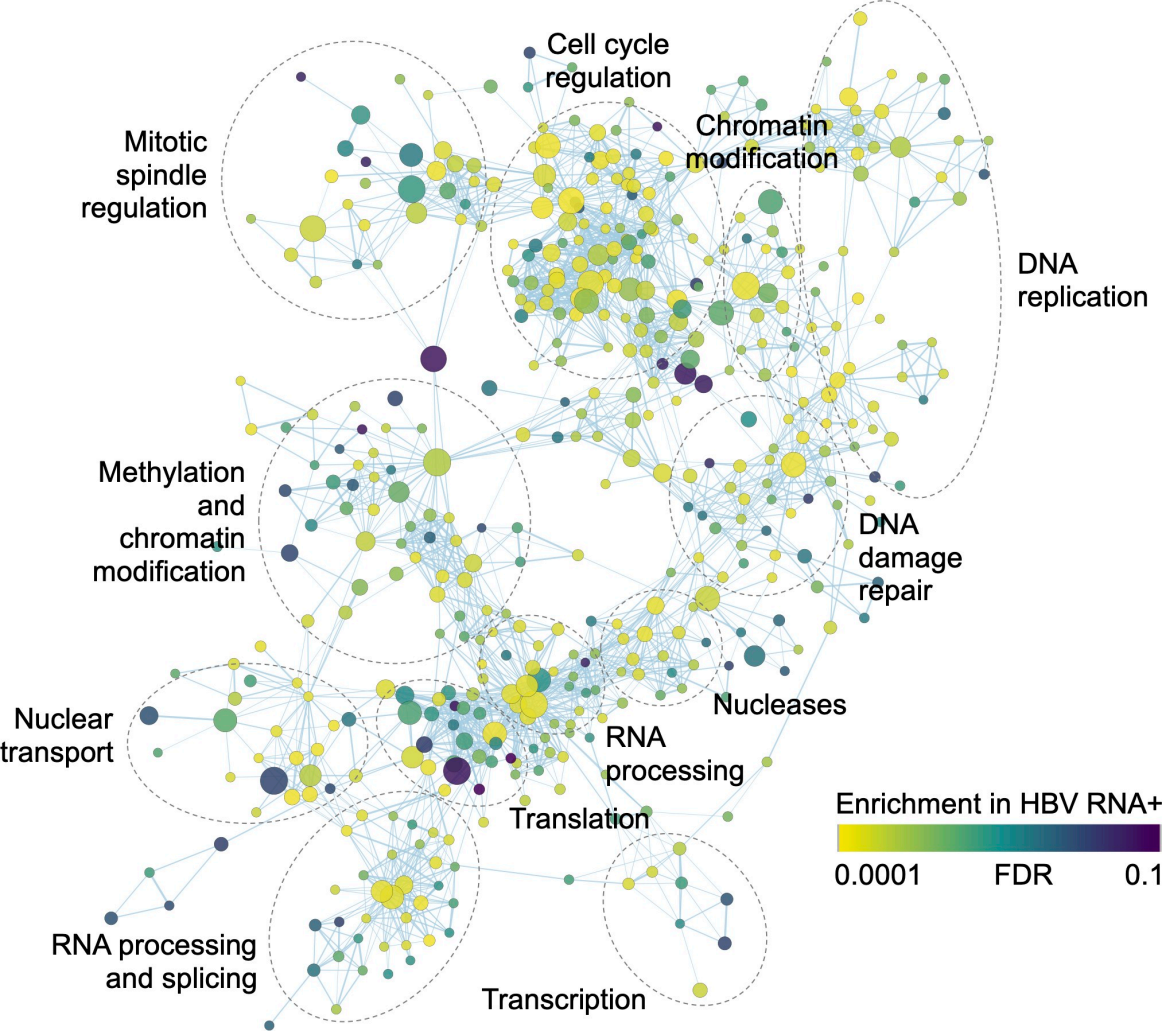
Schematic enrichment map of Gene Ontology Consortium gene sets enriched in HBV RNA positive TCGA tumors at a false discovery rate (FDR) < 10%, based on Gene Set Expression Analysis (*n* = 531 of 4,464), with gene set categories annotated. Circle size is proportional to gene set size. HBV, hepatitis B virus; TCGA, The Cancer Genome Atlas.

Next, we evaluated the effect of HBV RNA status on gene transcription, via gene set expression analysis of RNA-Seq gene level read counts from HCC tumors in the TCGA dataset. We found that a wide variety of canonical gene sets were enriched in HBV RNA positive tumors at a false discovery rate (FDR) of less than 10% (Fig 3). These included gene sets involved in multiple cellular processes, including cell cycle regulation, DNA replication, transcription, translation and DNA damage repair. We also found that gene sets upregulated in several previously published HCC subclasses that are felt to constitute a proliferative HCC phenotype were similarly enriched, including Boyault subclass G1-G3, Hoshida subclass S2, Lee subclass A and Chiang “proliferation” subclass HCC (S1 Fig) [17–21]. Of note, most enriched gene sets were enriched only in HBV RNA positive tumors; for example, all Gene Ontology Consortium gene sets enriched at FDR < 10% (531 of 4,464) were enriched in HBV RNA positive tumors, and none were enriched in HBV RNA negative tumors.

**Fig 4:**
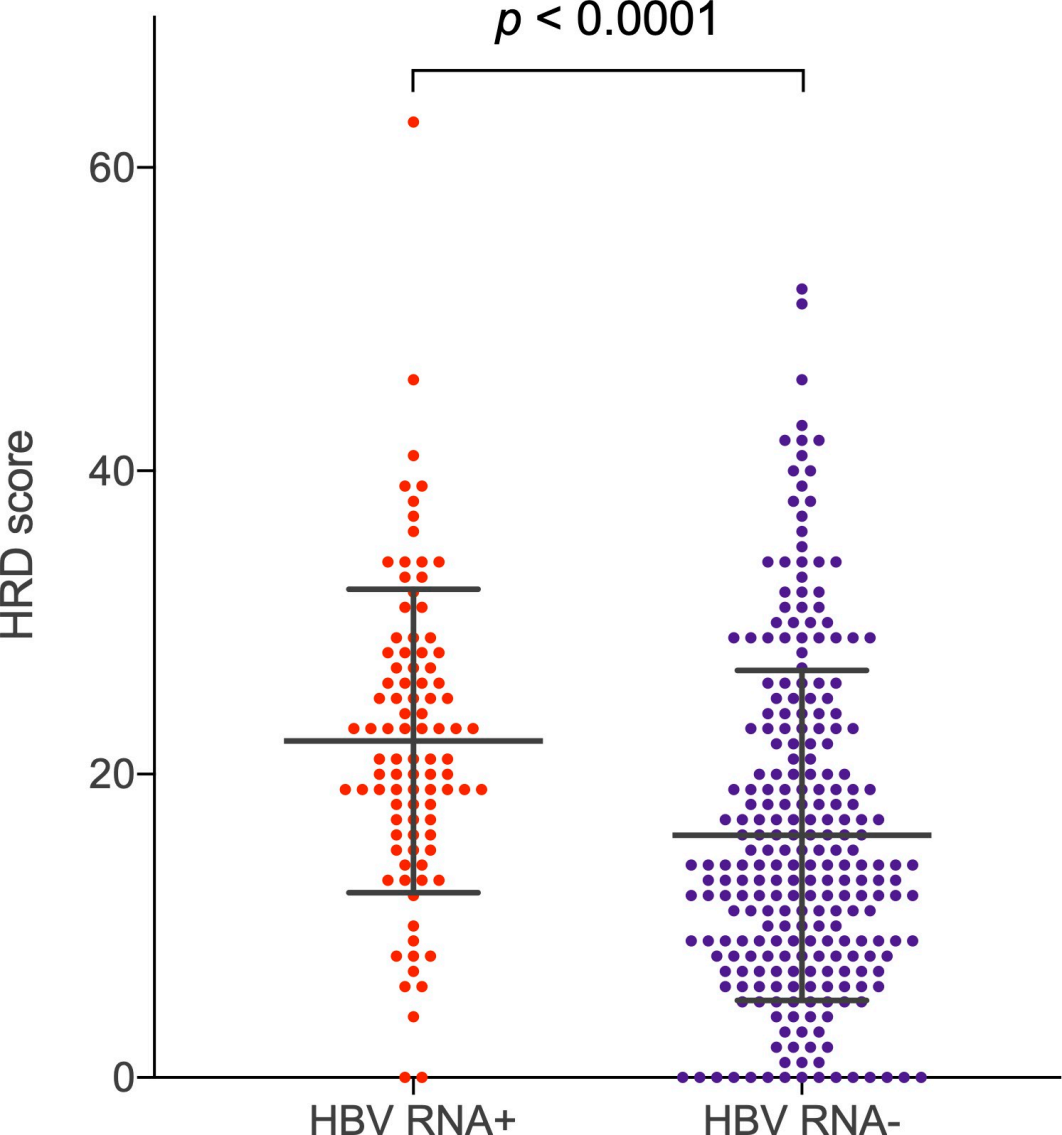
Homologous recombination deficiency (HRD) score for TCGA tumors, by HBV RNA status. HRD score was calculated as the sum of three independent HRD measures: Large-scale state transitions, loss of heterozygosity and telomeric allelic imbalance. Error bars show mean ± standard deviation. *p* value from Mann-Whitney test is shown. HBV, hepatitis B virus; TCGA, The Cancer Genome Atlas.

To further investigate the enrichment of multiple DNA damage repair gene sets in HBV RNA positive tumors, we evaluated the effect of HBV RNA status on three-biomarker homologous recombination deficiency (HRD) score and tumor mutational burden, hypothesizing that these markers of genomic instability might also be elevated in HBV RNA positive tumors. We found that three-biomarker HRD scores were significantly higher in HBV RNA positive tumors, in the subset of 348 TCGA tumors for which these HRD scores were available (mean HRD score 22.2 for HBV RNA positive tumors vs. 16.0 for HBV RNA negative tumors, *P* < 0.0001; Fig 4). Additionally, compared to HBV RNA negative tumors, there were substantially fewer HBV RNA positive tumors with a low or zero HRD score. Because *TP53* mutations have previously been associated with increased three-biomarker HRD score in multiple cancer types [22], we also investigated the effect of *TP53* mutation status on HRD score, and found that the presence of a nonsynonymous somatic *TP53* mutation was significantly associated with higher HRD score (mean HRD score 23.0 for *TP53* mutant tumors vs. 15.5 for *TP53* wildtype, *P* < 0.0001). When evaluating the effect of both HBV RNA and *TP53* mutation status on HRD score, we found that tumors which were both HBV RNA negative and *TP53* wildtype had significantly lower HRD scores compared to tumors with other permutations of these traits (mean HRD score 14.1 for HBV RNA negative/*TP53* wildtype vs. 20.6 for HBV RNA positive/*TP53* wildtype, 22.1 for HBV RNA negative/*TP53* mutant and 24.5 for HBV RNA positive/*TP53* mutant, *P* < 0.0001 by one-way ANOVA and *P* = 0.0002 or less by Tukey-Kramer post hoc test for pairwise comparisons between HBV RNA negative/*TP53* wildtype and all other groups; S2 Fig). We found no significant association between HBV RNA status and tumor mutational burden.

**Fig 5:**
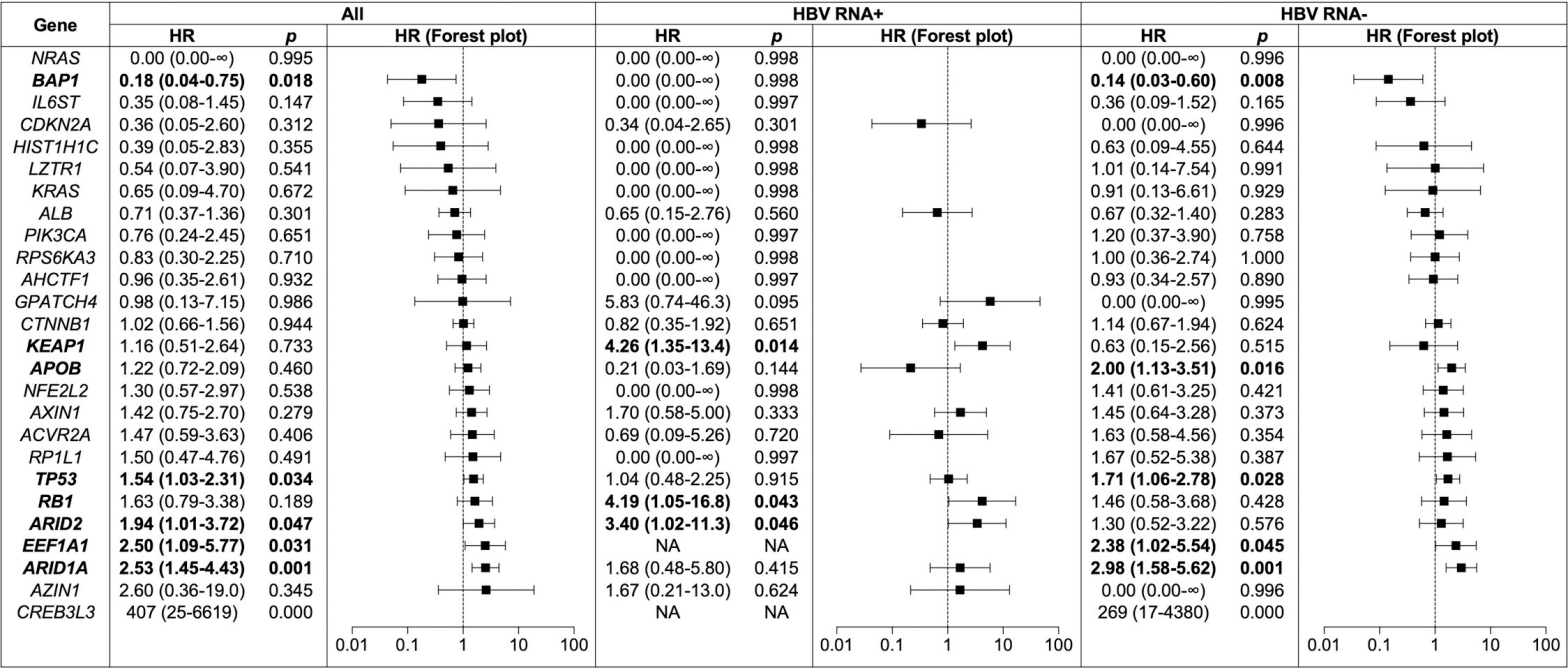
Hazard ratios and Forest plots of the prognostic effect of nonsynonymous somatic mutations in the 26 significantly-mutated genes identified in the TCGA hepatocellular carcinoma study on overall survival for HBV RNA positive, HBV RNA negative and all patients in the combined TCGA and ICGC cohort. Hazard ratios with 95% confidence intervals and *p* values from Cox multivariable regression analysis (covariates: HBV RNA status, mutation status, age, sex, grade, stage and cohort [TCGA or ICGC]) are shown. Error bars show 95% confidence intervals. Bolded values are those where the combination of mutation and HBV RNA status had a significant effect on overall survival. HBV, hepatitis B virus; TCGA, The Cancer Genome Atlas; ICGC, International Cancer Genome Consortium.

**Fig 6:**
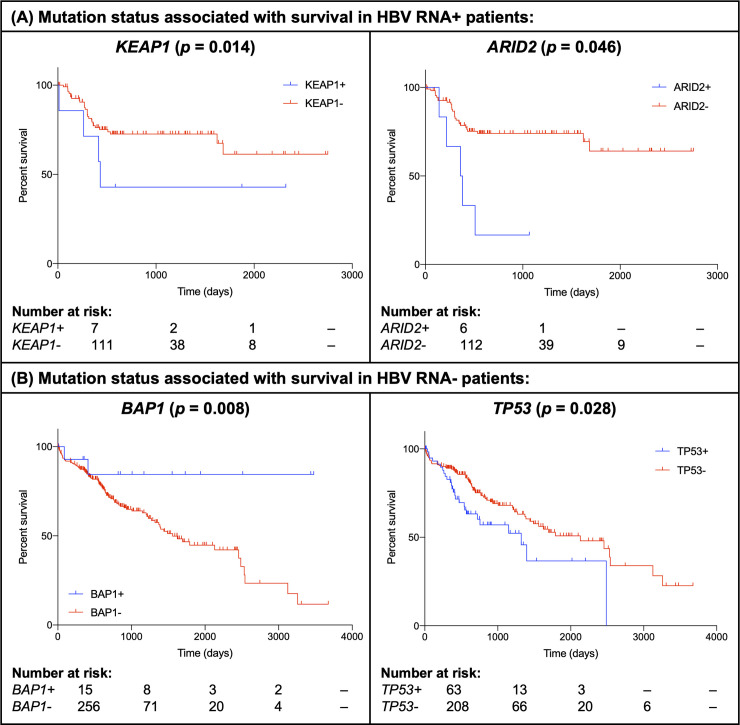
Kaplan-Meier survival curves illustrating the effect of selected nonsynonymous somatic mutations on overall survival in (A) HBV RNA positive patients and (B) HBV RNA negative patients in the combined TCGA and ICGC cohort. *p* values from Cox multivariable regression analysis (covariates: HBV RNA status, mutation status, age, sex, grade, stage and cohort [TCGA or ICGC]) are shown. HBV, hepatitis B virus; TCGA, The Cancer Genome Atlas; ICGC, International Cancer Genome Consortium.

Finally, we evaluated the effect of HBV RNA status on survival using Cox multivariable regression modeling, and found that HBV RNA status was associated with several mutation-specific survival differences (Figs 5 and 6). Examples include the tumor suppressor *BAP1*, where nonsynonymous somatic mutations were associated with increased survival across the entire cohort (*P* = 0.018)–an effect driven primarily by the association of *BAP1* mutations with survival benefit in HBV RNA negative patients (*P* = 0.008), as the vast majority of *BAP1* mutations in the cohort (20 of 21) were found in HBV RNA negative tumors. *BAP1* was the only gene in our study for which mutations were associated with a survival benefit. In contrast, nonsynonymous somatic mutations in several other genes were associated with diminished survival, including *KEAP1*, where mutations were associated with worse survival in HBV RNA positive patients, and *TP53*, where mutations were associated with worse survival in HBV RNA negative patients and across the entire cohort (*P* < 0.04 for all).

## Discussion

Despite recent advances in HCC genomics, much remains unclear, particularly with respect to HBV-related HCC. To our knowledge, this is the first study of HBV-related HCC where HBV RNA measurement in tumor tissue was used as the sole determinant of tumor HBV status, and the largest study of HBV-related HCC to have considered tumor HBV RNA measurement; prior studies have either based the assessment of HBV status partially or solely on clinical data such as reported HBV history or viral serologies, and, if tumor HBV RNA measurement was performed, utilized a smaller cohort. Here, we report that tumor HBV RNA measurement defines a unique subset of HCC characterized by distinct clinical and molecular characteristics.

In our cohort, we found that HBV RNA positive HCC was associated with similar clinical characteristics as reported in prior studies of HBV-related HCC, including male gender, Asian ethnicity and younger age and higher tumor grade at diagnosis, although the overall prevalence of HBV RNA positivity in our cohort, 28.2%, was slightly higher than the 22.4% reported for the core TCGA dataset [6,7]. We also found that HBV RNA positive HCC was associated with enriched transcription of gene sets involved in a wide variety of cellular pathways, as well as gene sets upregulated in the proliferative HCC phenotype associated with greater chromosomal instability and previously characterized by Boyault, Chiang, Hoshida, Lee and their colleagues [17–21]. To our knowledge, the association between HBV infection and all four of these proliferative HCC subclasses has not previously been reported.

Further, we found that for the 225 HBV clinical annotation positive patients in the TCGA dataset (i.e., those annotated as having a clinical history of either hepatitis B or hepatitis B surface antigen [HBsAg] positivity), 93 had HBV RNA positive HCC while 132 had HBV RNA negative HCC. This discrepancy between HBV clinical annotation and tumor HBV RNA status in a substantial subset of patients was unexpected. While inaccurate clinical annotation of HBV status is one possible explanation for this, another is that a subset of patients with HBV-related HCC may lack intrahepatic HBV integration or HBV gene expression–perhaps related to their having received antiviral treatment or to seroclearance of hepatitis B e antigen (HBeAg), which can be associated with a reduction in circulating HBV DNA out of proportion to reduction in circulating HBsAg levels [23]. As noted earlier, we also found that genes whose mutation rates depended significantly on HBV RNA status overlapped minimally with those whose mutation rates depended significantly on HBV clinical annotation status. Taken together, these findings suggest that HBV-related HCC may constitute a genomically heterogeneous group, within which tumors with HBV RNA positivity harbor important genomic differences compared to those without, and for which tumor segmentation based on HBV RNA status may be able to yield meaningful insights into this heterogeneity. Similar heterogeneity may also exist within viral subsets of other virus-associated tumor types, such as head and neck squamous cell carcinoma or gastric cancer.

A recent study found that HBV insertions can cause alterations in *CCNA2* and *CCNE1*, which encode the cell cycle regulators cyclin A2 and cyclin E1, respectively, and that these alterations are associated with a more aggressive HCC phenotype, termed cyclin-driven HCC, which is defined by a unique pattern of frequent chromosomal structural rearrangements consistent with a break-induced replication mechanism [24]. Our finding that HBV RNA positive HCC is associated with a significantly higher three-biomarker HRD score is consistent with this. The enriched transcription of multiple DNA damage repair pathway gene sets–including genes involved in mismatch, base excision and nucleotide excision repair–in HBV RNA positive tumors could also be consistent with this, as these pathways could potentially be upregulated as a compensatory mechanism in the setting of replication stress.

Additionally, we found that nonsynonymous somatic mutations in several genes significantly affected overall survival in our cohort, even after controlling for covariates. Of these, the only gene where mutations were associated with a survival benefit was *BAP1*, in HBV RNA negative patients. (*BAP1* mutations were present nearly exclusively in HBV RNA negative tumors in our cohort; the sole HBV RNA positive tumor harboring a *BAP1* mutation had a relatively low HBV RNA read count of 2.84 HBV reads per million human reads, placing it in the lowest decile of HBV RNA read counts among HBV RNA positive tumors.) BAP1 is a deubiquitinase and tumor suppressor that, in human liver cell models, appears to regulate chromatin accessibility and gene expression [25]. In other tumor types, *BAP1* mutations have been associated with both increased and decreased survival: while *BAP1* expression loss and germline mutations are associated with survival benefit in malignant pleural mesothelioma [26,27], they are linked to worse survival in uveal melanoma and clear cell renal cell carcinoma [28]. Thus, although we did not find that HBV RNA status itself was associated with survival, it did interact with specific mutations so as to alter differential survival outcomes, including *BAP1*, where mutations in HBV RNA negative patients were associated with increased survival. This–together with the finding that *BAP1* mutation rates were significantly higher, and *BAP1* mutations almost exclusively present, in HBV RNA negative tumors–suggests that *BAP1* mutant HCC could represent an HCC subtype characterized by lack of HBV activity in tumor and improved outcomes. While it is unclear why our cohort exhibited a near-complete lack of overlap between HBV RNA positive tumors and *BAP1* mutant tumors, two intriguing possibilities exist. First, recent evidence suggests that BAP1 plays an anti-apoptotic role in liver tissue and that *BAP1* mutant HCC is associated with PKA pathway dysregulation [25,29]; HBV infection in HCC may substitute directly for one of these functions and thus alleviate selective pressure that might otherwise have favored BAP1 loss. Second, BAP1 itself is known to interact with BRCA1; thus, HBV effects on HRD may render HBV RNA positive HCC more vulnerable to genomic instability in the presence of BAP1 loss and disfavor the incidence of that mutation.

Finally, we found that mutations in several genes were associated with worse survival, including *KEAP1* mutations selectively in the limited subset of 7 HBV RNA positive patients found to harbor these mutations. KEAP1 is a redox sensing protein that interacts with NRF2 as part of the oxidative stress response pathway. As discussed earlier, we found a nonsignificant trend towards *NFE2L2*, which encodes NRF2, being mutated almost exclusively in HBV RNA negative tumors. Taken together, these findings–that, in our cohort, HBV RNA positive tumors were characterized by a near-complete absence of *NFE2L2* mutations and an association between *KEAP1* mutations and diminished survival in the limited subset of patients harboring these mutations–could suggest a functional distinction in the manner in which the oxidative stress response pathway is dysregulated in HBV-mediated tumorigenesis compared to non-HBV HCC.

The finding that HBV RNA positive tumors harbored a substantial majority of differentially mutated genes and differentially expressed gene sets in our study was unexpected. As discussed earlier, tumor mutational burden did not vary significantly based on HBV RNA status, excluding higher overall mutation burden in HBV RNA positive tumors as the cause of this phenomenon. We also considered the possibility that HBV RNA negative tumors might comprise multiple genotypic subgroups, such that comparison of all such tumors as a single group could mask important between-subgroup variation, but principal component analysis of normalized RNA-Seq gene level read counts was inconsistent with this (S3 Fig). Thus, while the cause of this phenomenon remains unclear, one possibility is that HBV exerts a specific genotoxic stress which is independent of other tumor biology characteristics and possesses its own separate heterogeneity.

Our study has several limitations. First, we used tumor HBV RNA measurement as a proxy for viral activity, instead of measuring this more directly (e.g., by direct virion quantification in tumor tissue). We felt this was reasonable because transcription of HBV DNA into RNA is a key viral lifecycle event, and also because, as previously noted, we found excellent concordance between HBV RNA status and genomic evidence of HBV integration, another key lifecycle event. Second, although the threshold of 1 HBV RNA read per million human reads (RPM) used to define HBV RNA positive status was based on precedent for its use in prior studies [6,30], we could have selected a different threshold. However, using an alternative threshold of 0.5 or 2 RPM would not have changed our results substantially because tumor RPM scores were not clustered around the 1 RPM threshold. Third, our cohort was clinically and pathologically heterogenous, which may explain why some of our findings differ from prior published studies as previously discussed. Fourth, some of the previously noted associations between nonsynonymous somatic mutations and survival are based on limited sample sizes–e.g., 7 HBV RNA positive patients harboring *KEAP1* mutations were found to have significantly diminished survival–and thus may warrant cautious interpretation.

Our findings suggest possible avenues for future investigation. First, the association between HBV RNA positive HCC and elevated three-biomarker HRD score has not been previously reported and raises the question of whether these tumors may be amenable to HRD-targeted therapies. In other tumor types, three-biomarker HRD scores of 42 or greater have been associated with sensitivity to the PARP inhibitor niraparib (in recurrent ovarian cancer) [31] and platinum chemotherapy (in triple-negative breast cancer) [32]. While the HRD score threshold of 42 used in these studies is substantially higher than the median HRD score of 22 in our HBV RNA positive cohort, wide variation in HRD scores exists across tumor types and lower HRD score thresholds have been reported to be prognostic in other tumor types, such as prostate cancer, where an HRD score threshold of 20 has been associated with potential response to HRD-targeted therapies [33]. Also, while a prior phase II study of temozolomide and the PARP inhibitor veliparib in advanced HCC failed to show a survival benefit (ClinicalTrials.gov identifier NCT01205828), that study did not use tumor HBV status as a patient selection criterion [34]. Second, our results raise the question of to what degree tumor HBV RNA status may be concordant with serum markers of HBV infection such as serologies and viral load, and whether there may be an expanded role for direct measurement of tumor HBV RNA–e.g., via liver biopsy–in the pretreatment setting.

In conclusion, we report here that HCC tumors with genomic evidence of HBV activity constitute a distinct HCC subset characterized by specific differences in nonsynonymous somatic mutations, gene set expression, three-biomarker HRD score and mutation-specific survival. These differences may provide a rationale for future therapeutic trials, including trials of HRD-targeted therapies, in this unique patient subset. They also suggest that tumor segmentation based on genomic evidence of HBV activity could yield meaningful insights into the heterogeneity of HBV-related HCC, including with respect to the findings of prior therapeutic trials (for example, prior trials of multikinase inhibitors in advanced HCC have yielded discordant results regarding the effect of HBV status on survival [35,36], a possible explanation for which is that intratumoral viral activity may have been an unmeasured confounder). Further studies are needed to validate our findings in a larger cohort, and the questions raised here suggest that there is a critical need for an integrated genomic and virologic characterization of HBV-related HCC.

## Materials and methods

### Study cohort

We obtained genomic data for 439 histologically confirmed HCC tumors from two public databases, The Cancer Genome Atlas (TCGA; *n* = 371) and the International Cancer Genome Consortium (ICGC; *n* = 68). ICGC tumor data were obtained from the LIRI-JP dataset, as this was the only ICGC dataset for which tumor RNA-Seq data were publicly available. This study was not classified as human subjects research and was thus exempt from institutional review per the guidelines of the University of California, San Francisco, Institutional Review Board.

### HBV RNA measurement

We used GATK PathSeq (Broad Institute, Cambridge, MA) to quantify the number of HBV RNA reads present in RNA-Seq data for each tumor [37,38]. This software tool detects and quantifies microbial genomic sequences in host genomic data by filtering out low complexity, low quality and duplicate reads, then performs computational subtraction of human reads and aligns the remaining reads against a database of pathogen reference genomes, including HBV, using the BWA-MEM alignment algorithm. GATK PathSeq and its predecessor, PathSeq, have been used in multiple prior genomic studies of HCC and other cancer types [6,30]. We classified tumors as HBV RNA positive if at least 1 HBV RNA read was detected per million human RNA reads, selecting this threshold given precedent for its use in detecting HBV and other oncoviruses in prior studies [6,30]. All other tumors were classified as HBV RNA negative. We then investigated the association between HBV RNA status and patient characteristics, nonsynonymous somatic mutations, gene set expression, homologous recombination deficiency score and mutation-specific survival.

### Clinical and genomic data

Patient clinical characteristics were obtained from the TCGA and ICGC clinical databases (including, for TCGA tumors, the TCGA Clinical Data Resource) [39]. We obtained the nonsynonymous somatic mutations present in each TCGA and ICGC tumor from their respective data portals and considered only the following nonsynonymous mutation types: missense, frameshift, stop gain, start loss and in-frame deletion. For TCGA tumors, for which RNA-Seq gene level read counts normalized using the FPKM-UQ method were available, we also used Gene Set Expression Analysis software (GSEA; Broad Institute, Cambridge, MA) to evaluate gene set expression [40]. Additionally, we obtained previously defined three-biomarker homologous recombination deficiency (HRD) scores–calculated as the sum of three independent HRD measures: large-scale state transitions, loss of heterozygosity and telomeric allelic imbalance–for 348 TCGA tumors [22], and data regarding genomic evidence of HBV integration for the 193 tumors in the TCGA core dataset for which both integration and RNA-Seq data were available. Finally, we determined mutation-specific survival using survival and vital status data from the TCGA and ICGC clinical databases.

### Statistical analysis

We used χ^2^ and Mann-Whitney tests to compare the prevalence of clinicopathologic factors and Fisher’s exact test to compare the prevalence of nonsynonymous somatic mutations based on HBV RNA status. To compare mutation-specific survival differences, we constructed a Cox multivariable regression model where the covariates were HBV RNA status, mutation status, age, sex, grade, stage and cohort (TCGA or ICGC). For TCGA tumors, which also underwent gene set expression analysis and comparison of three-biomarker HRD scores, we used GSEA software to compute false discovery rate (FDR) *q* values and either the Mann-Whitney test or one-way ANOVA with the Tukey-Kramer post hoc test to compare HRD scores. Finally, we used the scikit-learn Python library to perform principal component analysis of RNA-Seq gene level read counts. All statistical tests were two-tailed and *P* values less than 0.05 were considered significant. Statistical analyses were performed using Excel 2019 (Microsoft, Redmond, WA), Prism 8 (GraphPad, San Diego, CA), scikit-learn, or R and its “survival” package.

## Supporting information

S1 Table (A) Proportion of HBV RNA positive and HBV RNA negative tumors harboring genomic evidence of HBV integration, in the core TCGA dataset of 193 tumors for which RNA-Seq data and genomic integration data were available. *p* < 0.0001 by χ^2^ test. (B) For HBV RNA positive tumors in the core TCGA dataset, breakdown by clinical annotation of either positive HBV history or positive HBsAg and by genomic evidence of HBV integration. (C) For HBV RNA negative tumors in the core TCGA dataset, breakdown by clinical annotation of either positive HBV history or positive HBsAg and by genomic evidence of HBV integration. (D) For all 371 tumors in the TCGA dataset, breakdown by clinical annotation of either positive HBV history or positive HBsAg and by HBV RNA status. HBV, hepatitis B virus; HBsAg, hepatitis B surface antigen; TCGA, The Cancer Genome Atlas.
(DOCX)

S2 Table Proportion of HBV RNA positive and HBV RNA negative tumors where paired normal liver tissue samples were HBV RNA positive or HBV RNA negative, for the subset of 50 TCGA tumors for which RNA-Seq data for paired normal liver tissue samples were available. *p* < 0.0001 by χ^2^ test. HBV, hepatitis B virus; TCGA, The Cancer Genome Atlas.
(DOCX)

S1 Fig Enrichment plots for four previously characterized gene sets associated with a proliferative HCC phenotype whose transcription was enriched in HBV RNA positive TCGA tumors, based on Gene Set Enrichment Analysis (GSEA). False discovery rate (FDR) *q* values from GSEA are shown. HCC, hepatocellular carcinoma; HBV, hepatitis B virus; TCGA, The Cancer Genome Atlas.
(TIF)

S2 Fig Homologous recombination deficiency (HRD) score for TCGA tumors, by HBV RNA status and nonsynonymous somatic TP53 mutation status. HRD score was calculated as the sum of three independent HRD measures: Large-scale state transitions, loss of heterozygosity and telomeric allelic imbalance. Error bars show mean ± standard deviation. *p* < 0.0001 by one-way ANOVA and *p* = 0.0002 or less by Tukey-Kramer post hoc test for pairwise comparisons between HBV RNA negative/*TP53* wildtype and all other groups. (Pairwise comparisons among HBV RNA positive/*TP53* mutant, HBV RNA positive/*TP53* wildtype and HBV RNA negative/*TP53* mutant groups were not significant by Tukey-Kramer post hoc test.) HBV, hepatitis B virus; TCGA, The Cancer Genome Atlas; mut, mutant; wt, wildtype.
(TIF)

S3 Fig Two-component principal component analysis (PCA) of normalized RNA-Seq gene level read counts from TCGA tumors. Explained variance was minimal (0.05) for each principal component. TCGA, The Cancer Genome Atlas.
(TIF)

S1 Data Comprehensive supplementary dataset for this study, including HBV RNA read counts and HBV RNA status for all TCGA and ICGC tumors, as well as other underlying data. HBV, hepatitis B virus; TCGA, The Cancer Genome Atlas; ICGC, International Cancer Genome Consortium.
(XLSX)
