# Supplementary material for: Tumor hepatitis B virus RNA identifies a clinically and molecularly distinct subset of hepatocellular carcinoma
Source: PLoS Comput Biol. 2021 Feb 9;17(2):e1008699. doi: 10.1371/journal.pcbi.1008699 (PMC7909678; doi:10.1371/journal.pcbi.1008699)
Supplement: S1 Table — (A) Proportion of HBV RNA positive and HBV RNA negative tumors harboring genomic evidence of HBV integration, in the core TCGA dataset of 193 tumors for which RNA-Seq data and genomic integration data were available. p < 0.0001 by χ2 test. (B) For HBV RNA positive tumors in the core TCGA dataset, breakdown by clinical annotation of either positive HBV history or positive HBsAg and by genomic evidence of HBV integration. (C) For HBV RNA negative tumors in the core TCGA dataset, breakdown by clinical annotation of either positive HBV history or positive HBsAg and by genomic evidence of HBV integration. (D) For all 371 tumors in the TCGA dataset, breakdown by clinical annotation of either positive HBV history or positive HBsAg and by HBV RNA status. HBV, hepatitis B virus; HBsAg, hepatitis B surface antigen; TCGA, The Cancer Genome Atlas. (DOCX) [file pcbi.1008699.s001.docx]

# S1 Table

|  | **HBV RNA+** | **HBV RNA-** |
| --- | --- | --- |
| **Genomic evidence of HBV integration** | 35 | 2 |
| **No genomic evidence of HBV integration** | 2 | 154 |

**S1 Table (A).** Proportion of HBV RNA positive and HBV RNA negative tumors harboring genomic evidence of HBV integration, in the core TCGA dataset of 193 tumors for which RNA-Seq data and genomic integration data were available. *p* < 0.0001 by χ^2^ test. HBV, hepatitis B virus; TCGA, The Cancer Genome Atlas.

| **HBV RNA+** | **Annotated as having either positive HBV history or positive HBsAg** | **Annotated as having neither positive HBV history nor positive HBsAg** | **Insufficient HBV/HBsAg annotation data for categorization** |
| --- | --- | --- | --- |
| **Genomic evidence of HBV integration** | 32 | 2 | 1 |
| **No genomic evidence of HBV integration** | 2 | 0 | 0 |

**S1 Table (B).** For HBV RNA positive tumors in the core TCGA dataset, breakdown by clinical annotation of either positive HBV history or positive HBsAg and by genomic evidence of HBV integration. HBV, hepatitis B virus; HBsAg, hepatitis B surface antigen; TCGA, The Cancer Genome Atlas.

| **HBV RNA-** | **Annotated as having either positive HBV history or positive HBsAg** | **Annotated as having neither positive HBV history nor positive HBsAg** | **Insufficient HBV/HBsAg annotation data for categorization** |
| --- | --- | --- | --- |
| **Genomic evidence of HBV integration** | 2 | 0 | 0 |
| **No genomic evidence of HBV integration** | 71 | 61 | 22 |

**S1 Table (C).** For HBV RNA negative tumors in the core TCGA dataset, breakdown by clinical annotation of either positive HBV history or positive HBsAg and by genomic evidence of HBV integration. HBV, hepatitis B virus; HBsAg, hepatitis B surface antigen; TCGA, The Cancer Genome Atlas.

|  | **Annotated as having either positive HBV history or positive HBsAg** | **Annotated as having neither positive HBV history nor positive HBsAg** | **Insufficient HBV/HBsAg annotation data for categorization** |
| --- | --- | --- | --- |
| **HBV RNA+** | 93 | 2 | 5 |
| **HBV RNA-** | 132 | 86 | 53 |

**S1 Table (D).** For all 371 tumors in the TCGA dataset, breakdown by clinical annotation of either positive HBV history or positive HBsAg and by HBV RNA status. HBV, hepatitis B virus; HBsAg, hepatitis B surface antigen; TCGA, The Cancer Genome Atlas.
