# Supplementary material for: Tumor hepatitis B virus RNA identifies a clinically and molecularly distinct subset of hepatocellular carcinoma
Source: PLoS Comput Biol. 2021 Feb 9;17(2):e1008699. doi: 10.1371/journal.pcbi.1008699 (PMC7909678; doi:10.1371/journal.pcbi.1008699)
Supplement: S2 Table — p < 0.0001 by χ2 test. HBV, hepatitis B virus; TCGA, The Cancer Genome Atlas. (DOCX) [file pcbi.1008699.s002.docx]

# S2 Table

|  | **HBV RNA+**  **(Paired Normal Liver Tissue)** | **HBV RNA-**  **(Paired Normal Liver Tissue)** |
| --- | --- | --- |
| **HBV RNA+ (Tumor)** | 5 | 2 |
| **HBV RNA- (Tumor)** | 2 | 41 |

**S2 Table.** Proportion of HBV RNA positive and HBV RNA negative tumors where paired normal liver tissue samples were HBV RNA positive or HBV RNA negative, for the subset of 50 TCGA tumors for which RNA-Seq data for paired normal liver tissue samples were available. *p* < 0.0001 by χ^2^ test. HBV, hepatitis B virus; TCGA, The Cancer Genome Atlas.
