# Supplementary figures and images for: Tumor hepatitis B virus RNA identifies a clinically and molecularly distinct subset of hepatocellular carcinoma
Source: PLoS Comput Biol. 2021 Feb 9;17(2):e1008699. doi: 10.1371/journal.pcbi.1008699 (PMC7909678; doi:10.1371/journal.pcbi.1008699)

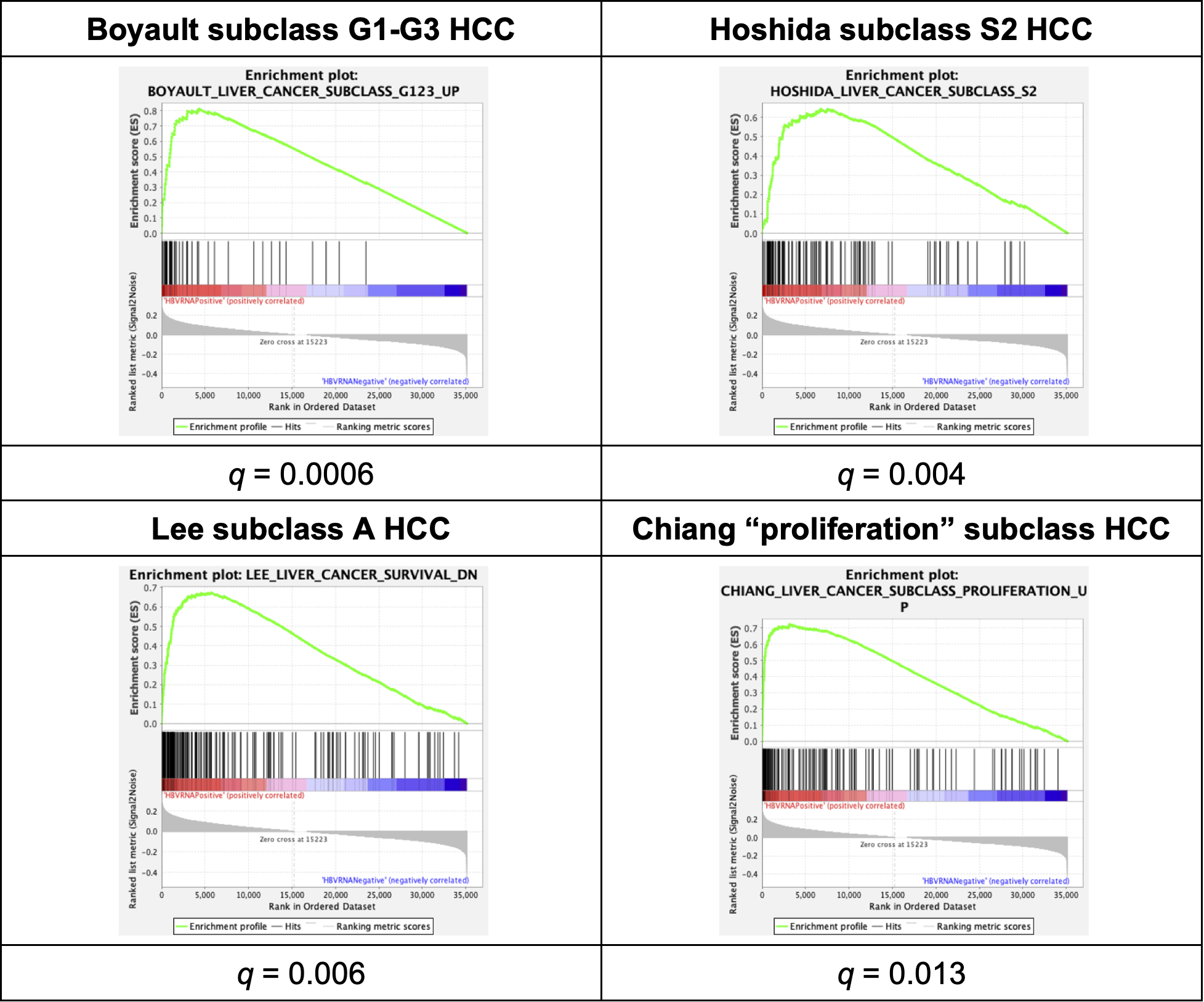

Supplement: S1 Fig — False discovery rate (FDR) q values from GSEA are shown. HCC, hepatocellular carcinoma; HBV, hepatitis B virus; TCGA, The Cancer Genome Atlas. (TIF) [file pcbi.1008699.s003.tif]

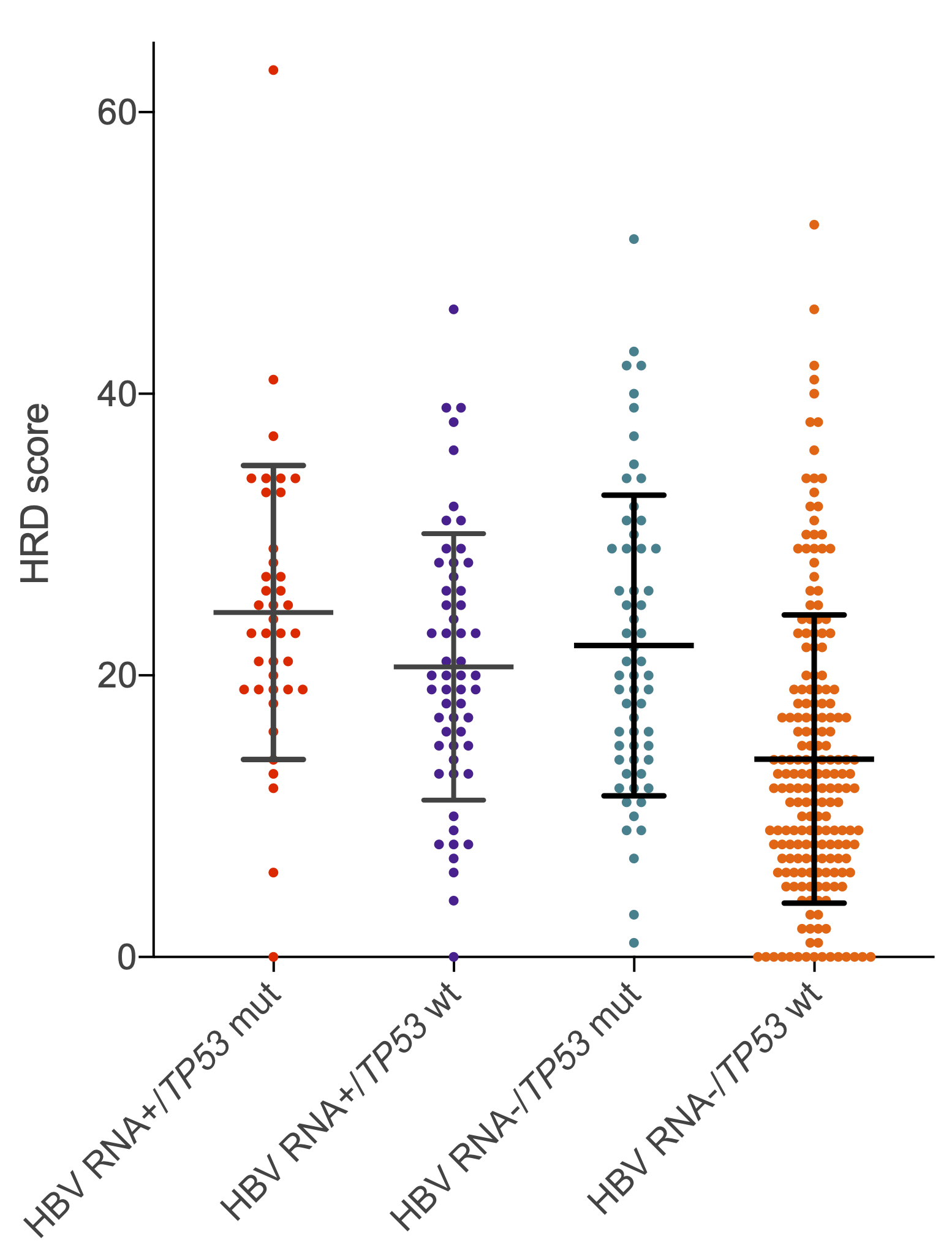

Supplement: S2 Fig — HRD score was calculated as the sum of three independent HRD measures: Large-scale state transitions, loss of heterozygosity and telomeric allelic imbalance. Error bars show mean ± standard deviation. p < 0.0001 by one-way ANOVA and p = 0.0002 or less by Tukey-Kramer post hoc test for pairwise comparisons between HBV RNA negative/TP53 wildtype and all other groups. (Pairwise comparisons among HBV RNA positive/TP53 mutant, HBV RNA positive/TP53 wildtype and HBV RNA negative/TP53 mutant groups were not significant by Tukey-Kramer post hoc test.) HBV, hepatitis B virus; TCGA, The Cancer Genome Atlas; mut, mutant; wt, wildtype. (TIF) [file pcbi.1008699.s004.tif]

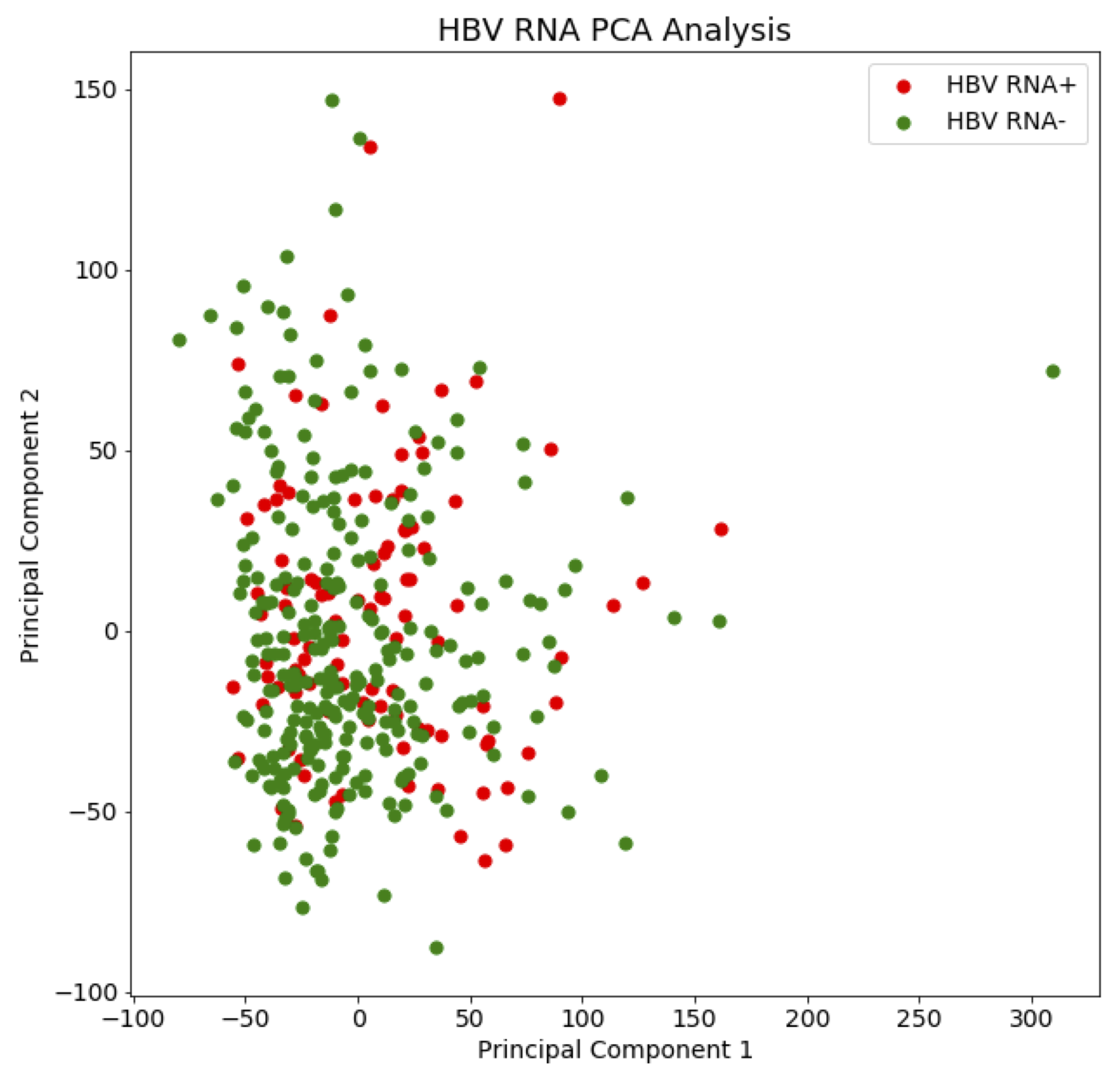

Supplement: S3 Fig — Explained variance was minimal (0.05) for each principal component. TCGA, The Cancer Genome Atlas. (TIF) [file pcbi.1008699.s005.tif]
